# A case of pediatric B-Lymphoblastic leukemia presenting with a t(9;12)(p24;q11.2) involving *JAK2* and concomitant *MLL* rearrangement with apparent insertion at 6q27

**DOI:** 10.1186/2050-7771-1-31

**Published:** 2013-11-25

**Authors:** Carlos A Tirado, David Shabsovich, Matthew DeNicola, Dinesh Rao, Lynn Yang, Rolando Garcia, Nagesh Rao

**Affiliations:** 1 Department of Pathology and Laboratory Medicine, David Geffen UCLA School of Medicine, Los Angeles, CA 90095, USA; 2 Department of Microbiology, Immunology, and Molecular Genetics, UCLA, Los Angeles, CA 90095, USA; 3 UT Southwestern Medical Center Department of Pathology, Dallas, Texas, USA

**Keywords:** *JAK2*, *MLL*, FISH, B-ALL

## Abstract

**Background:**

B-cell acute lymphoblastic leukemia (B-ALL) is the most common malignancy in pediatric patients and the leading cause of cancer-related death in children and young adults. Translocations of 9p24 involving *JAK2* (9p24) and gain-of-function mutations of *JAK2* with subsequent activation of the *JAK2* kinase have been described in several hematological malignancies including B-ALL. However, rearrangements involving *JAK2* are rare in B-ALL as only few cases have been described in the literature.

**Findings:**

Herein, we present a case of pediatric B-ALL whose conventional cytogenetics revealed an abnormal karyotype with a reciprocal translocation involving 9p24 (*JAK2*) and 12p11.2. Fluorescence in situ hybridization (FISH) studies using the RP11-927H16 Spectrum Green JAK2 probe on previously G-banded metaphases confirmed the involvement of *JAK2* in this rearrangement. Further FISH studies on the same previously G-banded metaphases using the LSI MLL probe helped to characterize an insertion of *MLL* into 6q27 as an additional abnormality in this karyotype. FISH studies performed on interphase nuclei also revealed an abnormal clone with *MLL* rearrangements in 23.6% of the nuclei examined as well as an abnormal clonal population with a deletion of the *5'IGH@* region in 88.3% of the nuclei examined.

**Conclusions:**

Rearrangements of 9p24 can result in constitutive activation of *JAK2*, and have been observed in B-ALL. Rearrangements of the *MLL* gene have also been described extensively in B-ALL. However, rearrangements of *MLL* with a partner at 6q27 and in conjunction with a translocation involving *JAK2* have not been previously described. This case pinpoints the importance of FISH and conventional cytogenetics to characterize complex rearrangements in which *JAK2* and *MLL* are involved. The therapeutic targeting of *JAK2* and *MLL* in cases like this may be prognostically beneficial.

## Introduction

Abnormalities involving *JAK2* (9p24) have been seen in B-ALL, but most often via point mutations involving the pseudokinase domain, R683 [1,2]. Rearrangements of 9p24, however, are rare, with only a small number of cases reported in the literature involving the following loci and partner genes: 22q11.2 (unknown gene), 12p13 (*ETV6*), 5p14.1 (*SSBP2*), 8p22 (*PCM1*), and 9p13.2 (*PAX5*) [1,3]. Activation of *JAK2* occurs via gene fusions encoding chimeric proteins in which the kinase domain of *JAK2* is fused to another cellular gene that provides a dimerization or oligomerization interface to the *JAK2* kinase domain, leading to constitutive activation [1-5]. This case pinpoints the fact that *JAK2* rearrangements may play an important role in the pathogenesis of lymphoblastic leukemias. To the best of our knowledge, this is one of the few cases with rearrangements of *JAK2* with chromosome 12p11.2 as well as rearrangements of *MLL* involving chromosome 6q27, both with unknown partner genes.

## Case presentation

A 13-year-old male presented with abdominal pain and fevers for three months. He was found to have leukocytosis (WBC 76.5x103/uL), anemia (Hgb 5.3 g/dL), and thrombocytopenia (platelet count 15.3x103/uL). Flow cytometry on peripheral blood revealed 94% blasts which expressed bright CD10, CD19, partial CD20, CD34, partial CD38, partial TdT, CD79a, and HLA-DR. A bone marrow biopsy showed a hypercellular marrow extensively involved (~95%) by sheets of lymphoblasts. These findings are consistent with a diagnosis of B-lymphoblastic leukemia. The patient was immediately started on induction chemotherapy with AALL0232 high-risk ALL chemotherapy protocol. A follow-up bone marrow biopsy on day 29 showed minimal residual disease (MRD). A normal karyotype was seen in all metaphase cells examined and loss of one copy of the 5′IGH@ was the only abnormality detected in 2.7% of the interphase nuclei studied. The patient subsequently was given treatment per clinical trial AALL0031 and achieved primary remission. Most recently, the patient received a successful allogeneic bone marrow transplant from a female donor.

## Methods

### Cytogenetics

Chromosome analysis was performed using standard cytogenetic techniques on bone marrow and peripheral blood, analyzing 20 metaphase cells. Karyotypes were prepared using Applied Imaging CytoVision software 2013 nomenclature [6].

### FISH

Fluorescence in situ hybridization (FISH) was performed on interphase nuclei and previously G-banded metaphases using the RP11-927H16 Spectrum Green JAK2 probe (Empire Genomics, 700 Michigan Ave, suite 200, Buffalo, NY 14203) and the following probes: Vysis LSI MLL Dual Color Break Apart Probe, Vysis LSI ETV6 Dual Color Break Apart, Vysis LSI ETV6(TEL)/RUNX1(AML1) ES Dual Color Translocation Probe Set and Vysis LSI IGH Dual Color, Break Apart Rearrangement Probe from Abbott Molecular (Des Plaines, Illinois 60018).

## Findings

### Cytogenetics

**Figure 1:**
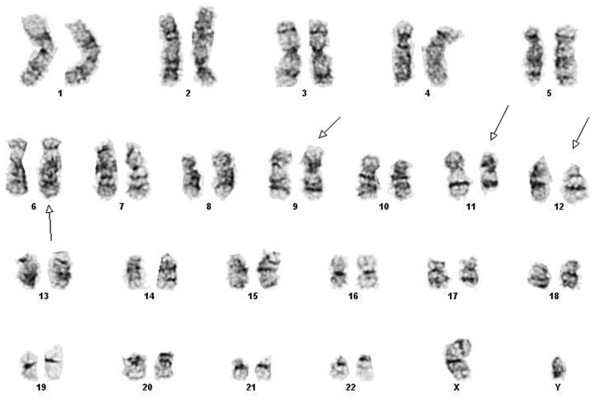
Abnormal karyotype seen on G-banded chromosomes in the bone marrow: 46,XY,ins(6;11)(q27;q23q23),t(9;12),(p24;p11.2)[5]/46,XY[15].

Chromosome analysis of the bone marrow showed 5 of 20 cells with an *MLL* insertion on 6q27 as well as a balanced translocation between 9p24 and 12p11.2 (Figure 1). The same abnormalities were seen on a karyotype performed on peripheral blood, though at a lower frequency (1 of 20 cells).

In light of the FISH findings the karyotype of the bone marrow of this patient was described as: **46,XY,ins(6;11)(q27;q23q23),t(9;12),(p24;p11.2)[5]/46,XY[15]**.

### FISH

**Figure 2:**
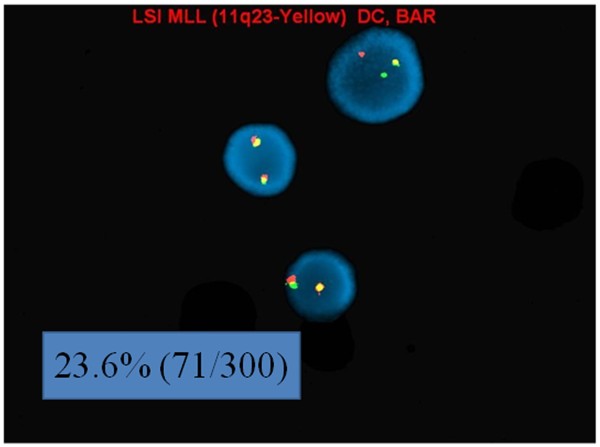
**Split *MLL *** **signals elucidated by FISH on interphase nuclei.**

**Figure 3:**
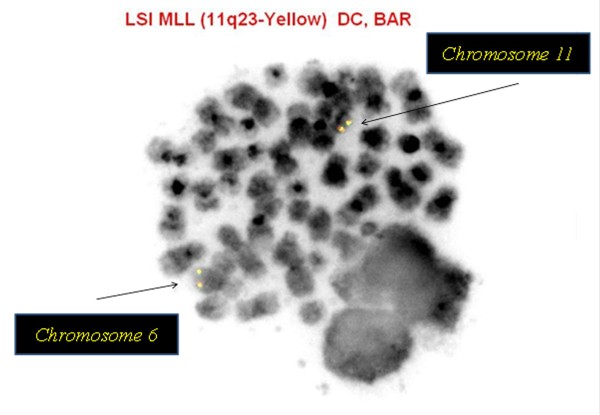
***MLL *** **insertion on 6q27 elucidated by FISH on previously G-banded metaphase chromosomes.**

**Figure 4:**
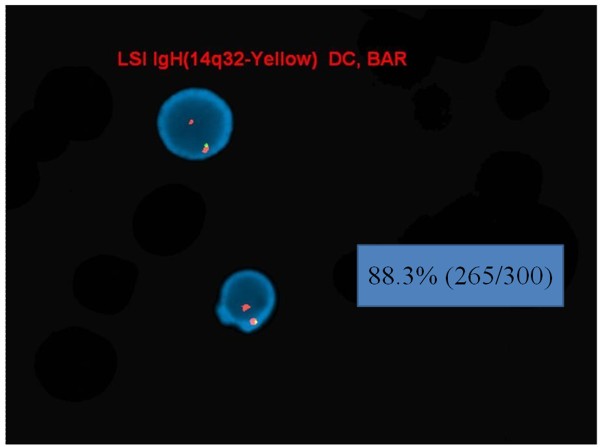
**Deletion of *5′ IGH@ *** **elucidated by FISH on interphase nuclei.**

FISH analysis using interphase nuclei showed *MLL* split signals in 23.6% (71/300) of the nuclei examined, suggestive of an *MLL* (11q23) gene rearrangement (Figure 2). However, FISH performed on previously G-banded metaphases also helped to identify two separate clonal populations with different *MLL* abnormalities: one with an *MLL* rearrangement mentioned above and one with an *MLL* insertion on chromosome 6q27 (Figure 3). Additionally, a deletion of the 5′ IGH@ region, corresponding to the variable segment of the IGH@ was seen in 88.3% (265/300) of the nuclei analyzed which may suggest a deletion of this region or an unbalanced rearrangement involving chromosome 14q32 (Figure 4).

**Figure 5:**
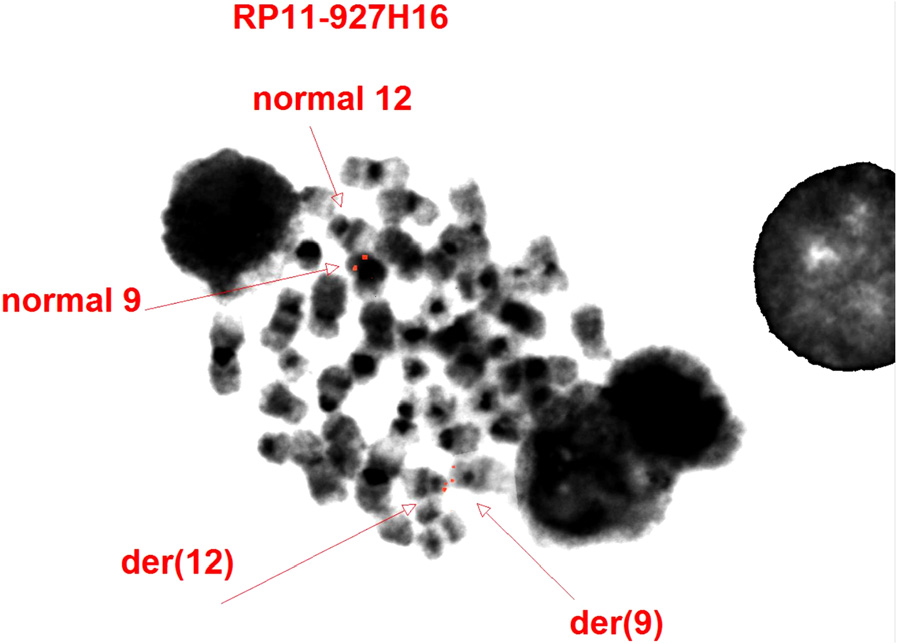
**FISH on metaphases using the BAC RP11-927H16 (*JAK2*) showed a signal on the normal copy of chromosome 9, a *JAK2 *** **signal on the short arm of chromosome 12, and a *JAK2 *** **signal on the derivative chromosome 9.**

**Figure 6:**
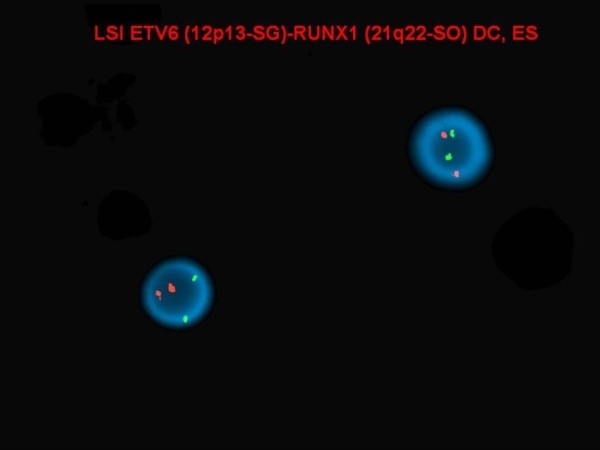
**There was no evidence of *ETV6/RUNX1 *** **translocation by interphase FISH.**

**Figure 7:**
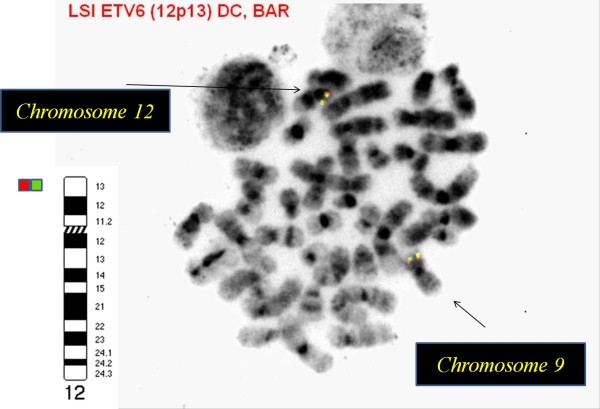
**There was no evidence of *ETV6 *** **rearrangements by metaphase FISH.**

FISH using the BAC RP11-927H16 probe (*JAK2*) showed a *JAK2* signal on the normal copy of chromosome 9, a *JAK2* signal on the short arm of chromosome 12, and a *JAK2* signal on the derivative chromosome 9 (Figure 5). Because there were no abnormalities involving *ETV6* (12p13), confirmed by using the Vysis LSI ETV6(TEL)/RUNX1(AML1) ES Dual Color Translocation Probe Set on interphase cells (Figures 6) and the Vysis LSI ETV6 Dual Color Break Apart on metaphase cells (Figure 7), the breakpoints on chromosome 12 were defined as 12p11.2 (Figure 7).

The constellation of these results was described as:

nuc ish(MLLx2)(5′MLL sep 3′MLLx1)[71/300].

nuc ish(ETV6,RUNX1)x2[300] .ish (5′ETV6,3′ETV6)x2(5′ETV6 con 3′ETV6x2).

nuc ish(3′IGH@x2,5′IGHx1) (3xIGH@con 5′IGH@x1) [265/300].

## Discussion

The findings in this case - *MLL* rearrangements, abnormalities of the *IGH@,* 12p abnormalities, and rearrangements of 9p24 involving the *JAK2* locus - have been previously described in B-ALL [1-3]. Abnormalities involving *IGH@* have only been recently identified as a biologically and clinically relevant sub-group of B-ALL [7]. However deletions of the 5′ *IGH@* region have not been well characterized in B-ALL in conjunction with *JAK2* rearrangements and *MLL* abnormalities. *JAK2* translocations have been reported in B-ALL, although at low frequencies. These B-ALL patients are most often male, present with hyperleukocytosis, respond poorly to chemotherapy, often relapse, and tend to have little to no cytogenetic abnormalities apart from those involving *JAK2*[1]. This fact may suggest that *JAK2* rearrangements play a driving role in the leukemogenesis of B-ALL.

*JAK2* translocations induce dimerization or oligomerization of *JAK2* without ligand binding, resulting in constitutive activation of *JAK2*-mediated tyrosine kinase pathways. It has been speculated that other cytogenetic abnormalities occurring in conjunction with *JAK2* rearrangements in B-ALL may recruit other altered tyrosine kinase pathways that in turn, lead to an inferior clinical outcome. A correlation has also been observed between *CRLF2* (cytokine receptor-like factor 2) overexpression and *JAK2* mutations, most likely because *CRLF2* is a *JAK*-binding, Box 1 motif-containing cytokine receptor. Increased expression of *CRLF2* independently has been correlated with a poor prognosis in B-ALL, and the synergistic effects of *CRLF2* overexpression and *JAK2* constitutive activation may play a major role in the leukemogenesis of the disease that can be prognostically considered and therapeutically targeted [8]. Similarly, even point mutations and rearrangements in the *CRFL2* gene have been reported to activate aberrant *JAK2* signaling [9].

While *JAK2* translocations are not common in lymphoblastic leukemia, it is clear that newly developed small molecular *JAK2* inhibitors such as TG101348 and TG10129 developed by TargetGen, Inc. show promising results in blocking the action of mutated *JAK2* in myeloproliferative disorders [2,10]. There are at least 10 different *JAK* inhibitors undergoing various phases of clinical trials [11] including a group of TKIs used for both MPDs and non-MPDs, namely MK-0457 (previously VX-680), that has had *JAK2* inhibitory action in MPD and reduced kinase activity in T315I-positive ALL and CML [2]. Lestaurtinib I(CEP-701), used mainly for myeloid malignancies, has also been used in a clinical trial to treat children with B-ALL [11]. However, among neoplasias dependent on tyrosine kinases, treatment with ATP-mimetic inhibitors has invariably resulted in the development of inhibitor resistance mutations [9]. A novel *JAK2* inhibitor, NVP_BVB808 (BVB808), has been used experimentally in mice xenografted with human B-ALL to recover E864K, Y931C, and G935R mutations within the kinase domain of *JAK2* that confer resistance to multiple *JAK2* enzymatic inhibitors [9]. In addition, treatment with inhibitors of heat shock protein 90 (HSP90) has now been used experimentally to overcome all three resistance mutations and potentially kill cells dependent on *JAK2*. However, development of new therapies that target the abnormal *JAK2* tyrosine kinase activity may benefit patients diagnosed with ALL presenting with *JAK2* rearrangements [9].

Structural abnormalities involving the *MLL* gene (11q23) with various partner genes have been reported in ALL in ~6% of cases, but an *MLL* insertion at 6q27 has not been reported to the best of our knowledge [12]. Herein, conventional and molecular cytogenetic metaphase analysis solely revealed an insertion of *MLL* on chromosome 6q27 with an unknown fusion partner gene; however, further molecular cytogenetic studies on interphase nuclei unveiled a second clonal population of cells harboring an *MLL* rearrangement. Inversion of *MLL* may, however, have followed rearrangements with chromosome 6 (as opposed to preceding it). Limited sample material prevented further molecular characterization. Furthermore, *MLL* insertions have been reported to result in chimeric fusion genes and are usually associated with a poor prognosis [3,12].

In short, our case highlights the importance of using multiple tools, namely conventional cytogenetic and molecular genetic analysis, to elucidate complex rearrangements involving *JAK2* and *MLL* genes. The detection and therapeutic targeting of *MLL* as well as *JAK2* abnormalities in cases of ALL may be prognostically beneficial as they may represent a distinct subtype of acute lymphoblastic leukemia. To the best of our knowledge, this study is the first reported case of a pediatric B-ALL that shows a concurrent *MLL* gene rearrangement with a *JAK2* translocation and deletion of the 5′ *IGH@* region. This case sheds light on the potential significance of *JAK2* and *MLL* as prognostic and therapeutic targets in lymphoblastic leukemias, and suggests further investigation to determine the benefits of the newly developed *JAK2* inhibitors against translocations involving *JAK2* in pediatric B-ALL*.*

## Competing interests

The authors declare that they have no competing interests.

## Authors’ contributions

CAT led the entire writing of the manuscript. DS wrote the first draft, conducted a survey for relevant literature, and edited and revised future drafts. MDN contributed with the discussion and reviewed the manuscript. RG edited the manuscript and helped to organize the figures. DR contributed with the clinical history. LY conducted all the cytogenetics, FISH, and bench work and interpretation of the results. NR edited and revised the final draft. All authors read and approved the final manuscript.
